# Zenker's peroral endoscopic myotomy for treating zenker's diverticulum in an elderly patient: a case report and literature review

**DOI:** 10.3389/fsurg.2026.1807255

**Published:** 2026-06-02

**Authors:** Xiao Fan, Jianhua Pang, Junmin Wang, Xinying Zhu, Li Jiao, Huan Ma, Xia Meng

**Affiliations:** 1 Department of Gastroenterology, Hebei Medical University Third Hospital, Shijiazhuang, Hebei, China; 2 Department of Endocrinology, Hebei Medical University Third Hospital, Shijiazhuang, Hebei, China

**Keywords:** dysphagia, endoscopic treatment, minimally invasive surgery, submucosal tunneling, zenker's diverticulum

## Abstract

**Background:**

Zenker's diverticulum (ZD) is a rare esophageal disorder, and traditional surgical interventions are associated with significant trauma and poor tolerance in elderly patients.

**Case summary:**

An 85-year-old female presented with a one-year history of progressive dysphagia, accompanied by regurgitation, coughing, and a 5 kg weight loss. Diagnostic evaluations, including barium esophagography, chest computed tomography (CT), and endoscopy, confirmed an upper esophageal ZD (3 cm in diameter).

**Treatment:**

The patient underwent submucosal tunneling endoscopic septum division (STESD), which is widely standardized in current literature as Zenker's peroral endoscopic myotomy (Z-POEM). Key procedural steps included mucosal incision, submucosal tunneling, complete septum myotomy, and mucosal closure using titanium clips. Postoperative recovery was uneventful, with no complications such as bleeding or perforation.

**Conclusion:**

Z-POEM demonstrates minimally invasive advantages, safety, and efficacy in treating ZD, particularly for elderly patients with high surgical risks. This technique represents a promising therapeutic option in this population.

## 1 Introduction

Zenker’s Diverticulum (ZD) is a rare upper esophageal pouch, predominantly observed in individuals aged over 60 years, with an estimated prevalence of 0.01%–0.11% (1). Anatomically, it arises from the herniation of the mucosa and submucosa through a muscular defect in the pharyngoesophageal junction (Killian's triangle), forming a pseudodiverticulum (2). Pathophysiologically, ZD is closely linked to cricopharyngeal muscle dysfunction, increased intraluminal pressure, and localized anatomical weakness, leading to repetitive mucosal trauma during swallowing and subsequent pouch formation (3).

Traditional surgical management, including open diverticulectomy or diverticulopexy, remains definitive but is associated with significant invasiveness, high complication rates (e.g., recurrent laryngeal nerve injury, fistula formation, and infection), and prolonged recovery, particularly unfavorable for elderly patients or those with cardiopulmonary comorbidities (4). Literature reports complication rates of 15%–30% and hospital stays exceeding 7 days for open procedures (1).

In recent years, submucosal tunneling endoscopic septum division (STESD), frequently standardized in recent literature as Zenker's peroral endoscopic myotomy (Z-POEM) (5), has gained attention for its minimally invasive profile and safety. This technique involves creating a submucosal tunnel to fully expose and transect the diverticular septum, preserving mucosal integrity to reduce perforation risk (<5%) while effectively relieving esophageal obstruction (6). Multiple studies confirm that Z-POEM achieves dysphagia resolution rates exceeding 90%, with mean hospitalization durations shortened to 3–5 days (7, 8).

Despite the growing application of Z-POEM, reports on its use in very elderly patients (e.g., those over 85 years) remain limited. While Z-POEM is technically established in younger, healthier cohorts, evidence remains sparse for its application in octogenarians with significant pre-existing frailty and multi-comorbidities. This case provides a unique clinical insight by demonstrating that for the extremely elderly, technical success must be integrated with a tailored, conservative perioperative strategy to achieve definitive outcomes while mitigating the high risks of aspiration and malnutrition. This article details the Z-POEM treatment of an 85-year-old patient with ZD, analyzing procedural nuances and clinical outcomes in conjunction with existing literature, aiming to provide insights into personalized management for high-risk elderly populations.

## 2 Case report

### 2.1 Patient information

The patient is an 85-year-old female admitted to the hospital on September 23, 2021, due to “dysphagia for 1 year, worsening over the past 3 months.” A year before admission, the patient experienced dysphagia without any apparent cause, particularly severe when consuming solid foods; symptoms partially alleviated upon drinking water. She did not receive standardized treatment. Over the last three months, the symptoms progressively worsened, accompanied by postprandial vomiting of undigested food and mucus, occasional acid regurgitation, and choking when drinking. She experienced a weight loss of 5 kg. The patient has no history of hypertension, diabetes, cardiovascular disease, or other gastrointestinal issues. She denies having any previous surgeries, trauma, or drug allergies.

### 2.2 Clinical presentation

Upon admission, the patient's vital signs were as follows: temperature 36.6 °C, heart rate 80 beats/min, respiratory rate 20 breaths/min, and blood pressure 133/80 mmHg. Physical examination showed no scleral icterus, and conjunctiva was not pale. Heart and lung auscultation revealed no abnormal sounds. The abdomen was flat and soft, with no tenderness, rebound pain, or rigidity. The liver and spleen were not palpable below the costal margin. Murphy's sign was negative; shifting dullness was negative; bowel sounds were four times per minute, and there was no edema in the lower extremities. Laboratory tests revealed hemoglobin at 112.90 g/L and hematocrit at 33.54%. Other routine blood tests, comprehensive biochemical panels, coagulation profiles, and preoperative infection screenings, including COVID-19 PCR, showed no abnormalities.

### 2.3 Diagnostic process

**Figure 1:**
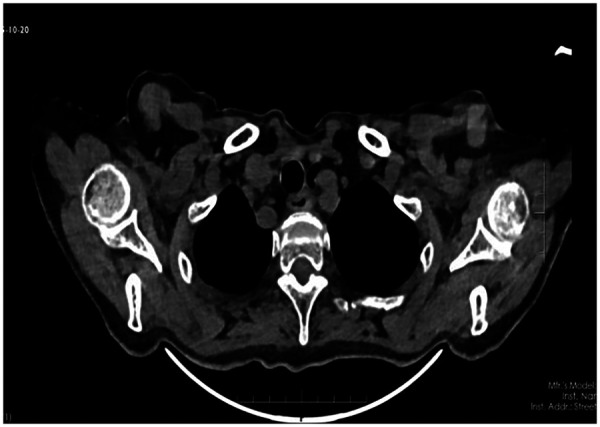
Chest CT scan.

**Figure 2:**
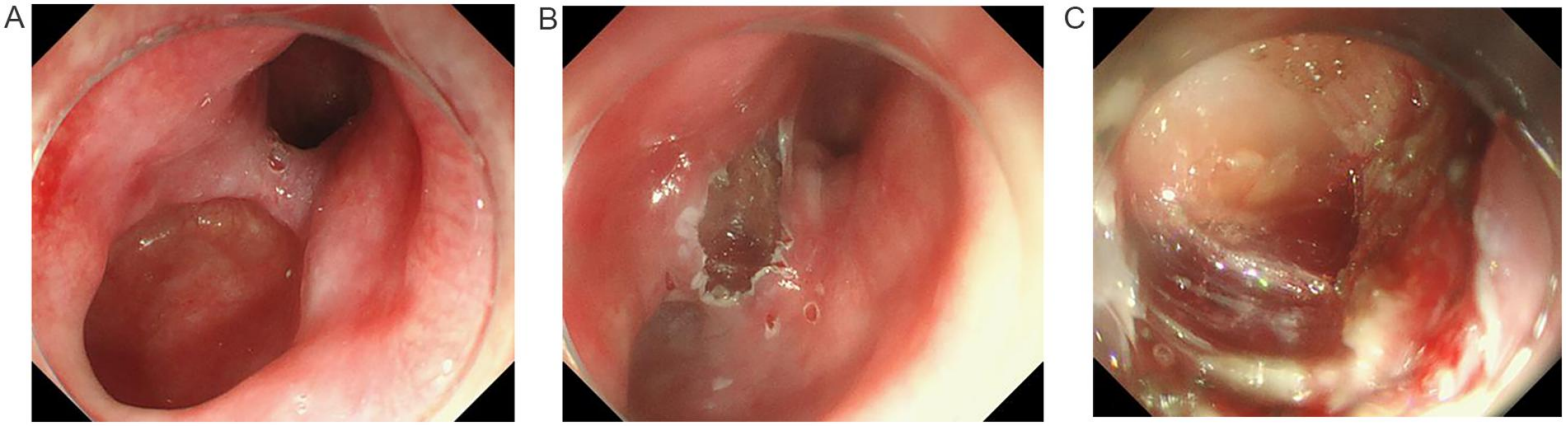
Surgical steps of zenker's peroral endoscopic myotomy (Z-POEM). **A**. Esophageal diverticulum; **B**. Creation of the tunnel; **C**. Transection of the diverticulum septum; **D**. Closure of the mucosal incision. Blue arrow: Diverticulum.

Thoracic CT scans at our hospital clearly demonstrated a pouch-like protrusion originating from the posterior wall of the upper esophagus at the level of the pharyngoesophageal junction, consistent with the diagnosis of Zenker's diverticulum (Figure 1). Cardiac ultrasound revealed a small amount of tricuspid regurgitation and reduced diastolic function of the left ventricle (E/A ratio < 1), with a normal left ventricular ejection fraction (LVEF). Ultrasounds of the liver, biliary system, pancreas, spleen, and kidneys showed no structural abnormalities; the portal vein diameter and blood flow velocity were within normal limits. Gastroscopy identified a large diverticulum measuring 3 cm in diameter at 15–16 cm from the incisors with smooth basal mucosa, without ulcers or active bleeding (Figure 2A). Combined with clinical history, imaging, and endoscopic results, the final diagnosis was Zenker's diverticulum.

### 2.4 Treatment methods

Upon completing the cardiopulmonary functional assessment and excluding surgical contraindications, the patient underwent a Z-POEM under general anesthesia on September 26, 2021. Surgical steps included: (1) under continuous carbon dioxide (CO₂) insufflation to minimize the risk of mediastinal emphysema and postoperative bloating, a submucosal injection of a mixture of glycerol fructose, methylene blue, and epinephrine was administered 2 cm proximal to the septum, forming a mucosal lift; (2) creation of a submucosal tunnel by a 1.5 cm longitudinal mucosal incision using a Dual knife (KD-650L; Olympus, Tokyo, Japan) powered by an electrosurgical unit (VIO 300D; ERBE, Tübingen, Germany) set to Endocut mode (Effect 1, Duration 4, Interval 1), and utilizing a transparent cap attached to the endoscope tip to facilitate submucosal dissection and ensure full, stable exposure of the septum within the tunnel (Figure 2B); (3) separation of the submucosal layer on both sides of the septum and complete transection of the muscular layer down to the base of the diverticulum, ensuring no residual muscle fibers (Figure 2C). Throughout these steps, the visual field within the tunnel was optimally maintained through dynamic endoscope rotation and controlled CO₂ insufflation; (4) electrocautery hemostasis using Coagulation mode, followed by closure of the mucosal incision using five titanium clips (Figure 2D). The total procedure time was 90 min, with the active operative time (from mucosal incision to final clip placement) being 55 min. No perforations or active bleeding occurred intraoperatively, and the surgery was successfully completed by an experienced endoscopist. Postoperative care included temporary cessation of oral intake, administration of intravenous rabeprazole 10 mg twice daily for acid suppression, ceftriaxone 1.0 g once daily for anti-inflammatory treatment, and nutritional support with 0.9% sodium chloride 200 mL and amino acids administered intravenously. The patient's condition remained stable postoperatively. After 72 h, liquid diet was resumed without discomfort; after observing tolerance to liquid diet for 72 h, gradual transition to soft foods commenced without symptoms such as hematemesis, chest pain, or fever.

Postoperative diagnosis: Upper esophageal diverticulum.

### 2.5 Treatment outcomes

The patient's postoperative recovery was smooth, with no chest pain, fever, hematemesis, or melena. A liquid diet started 72 h post-operation, and she was discharged from the hospital 12 days later. Follow-up one month post-surgery showed complete resolution of dysphagia, absence of acid regurgitation or choking, and a weight gain of 4 kg. One-year post-surgery telephone follow-up indicated complete resolution of dysphagia, absence of procedural complications, and a documented total weight gain of 3.6 kg since the procedure. Although our follow-up protocol initially included objective anatomical assessments, the patient and her family strongly refused further endoscopy or barium esophagography due to her advanced age and the perceived burden of additional hospital visits. Respecting their wishes, we relied on these robust clinical surrogate markers—sustained symptomatic relief and significant nutritional improvement—as indicators of clinical success.

## 3 Discussion

ZD is a rare but debilitating condition that predominantly afflicts the elderly population, leading to severe complications such as malnutrition and aspiration pneumonia if left untreated (9, 10). Historically, traditional open surgical interventions (e.g., diverticulectomy) were the standard of care; however, the literature extensively reports that they are associated with significant morbidity, prolonged hospitalization, and high complication rates, making them particularly prohibitive for frail, high-risk geriatric patients (3, 11). Consequently, the therapeutic paradigm has rapidly shifted towards minimally invasive endoscopic techniques. Among these, third-space endoscopy, specifically Z-POEM, has emerged in recent studies as a highly effective and safe alternative (12, 13). The present case of an 85-year-old patient contributes to the evolving evidence for Z-POEM by highlighting its feasibility and safety in the very elderly population. Although Z-POEM is increasingly recognized as a minimally invasive option, data on octogenarians and nonagenarians are still comparatively scarce. Our successful experience provides a valuable reference for managing such high-risk patients.

Li et al. (14) reported a new technique for treating esophageal diverticula using submucosal tunneling endoscopic septum division, which preserves mucosal integrity while significantly reducing the incidence of complications such as perforation and infection. Furthermore, complete transection of the septum minimizes the risk of diverticulum recurrence. Hernández et al. (15) provided preliminary evidence of the effectiveness and safety of third-space endoscopic techniques for esophageal diverticula treatment. To date, clinical observations and studies (12, 16, 17) have reported high surgical success rates, low complication rates, and favorable outcomes using Z-POEM for esophageal diverticula.

In the present case, the patient's symptoms of progressive dysphagia, regurgitation of undigested food, coughing, and significant weight loss were classic manifestations of an enlarging Zenker's diverticulum (1–3). The concern for potential complications such as aspiration and malnutrition, as evidenced by her 5 kg weight loss, underscored the necessity for intervention. Given the patient's advanced age and the associated risks of traditional surgery, a minimally invasive approach was imperative. Therefore, Z-POEM was chosen as a highly valuable and optimal alternative to mitigate the potential for severe postoperative complications in this vulnerable geriatric profile. One-year follow-up showed alleviation of dysphagia, acid regurgitation, vomiting, and coughing, with a 3.6 kg weight gain. Because of the patient's high age and limited mobility, she and her family declined further endoscopic or upper gastrointestinal imaging evaluations, resulting in lack of detailed post-treatment visual assessment.

The management of ZD has evolved from open surgery to less invasive endoscopic techniques, with third-space endoscopy emerging as a key innovation (14). Based on our procedural approach—specifically, creating a mucosal incision 2 cm proximal to the septum to establish a submucosal tunnel—the Z-POEM technique utilized in this case aligns precisely with what is currently standardized in the literature as Z-POEM (13, 18). It is crucial to distinguish this from Zenker's per-oral endoscopic septotomy (Z-POES). While Z-POES also operates within the third space, it involves an over-the-septum mucosotomy directly at the diverticular bridge and is typically reserved for shorter septa (< 2 cm) (19). Given our patient's large diverticulum (3 cm), the classic Z-POEM approach (proximal incision) was explicitly chosen to secure a longer, more reliable mucosal flap for safe closure.

Furthermore, the advantages of this third-space approach (Z-POEM) become particularly evident when compared to traditional flexible endoscopic septotomy (FES). Unlike classical FES, which involves a direct, full-thickness incision of the mucosa and septum, Z-POEM preserves mucosal integrity. This provides a significant advantage by theoretically reducing the risk of micro-perforation and subsequent mediastinitis—a critical consideration in vulnerable elderly patients (13, 14). Notably, while traditional FES heavily relies on a diverticuloscope to stabilize the septum and protect the posterior wall, our Z-POEM approach did not utilize one (19). In Z-POEM, the submucosal tunnel itself acts as an excellent natural working space (17). A standard transparent cap on the endoscope tip was sufficient to provide clear visualization and precise tissue traction. The mucosal flap inherently protects the surrounding structures, rendering a bulky diverticuloscope unnecessary and allowing for more agile maneuvers within the third space. Moreover, recent comparative evidence underscores that while FES may offer shorter procedure times, Z-POEM allows for a more controlled and complete myotomy of the cricopharyngeal muscle. A recent multicenter case-matched study by Provenzano et al. demonstrated that Z-POEM achieves superior clinical success and significantly lower recurrence rates compared to transoral septotomy (FES), primarily because the protective mucosal flap allows the endoscopist to extend the myotomy safely down to the base without the fear of transmural perforation (5). However, the primary disadvantages of Z-POEM are its technical complexity and the associated learning curve, requiring expertise in third-space endoscopy (20). While this may suggest longer procedure times initially, studies have shown that with experience, the duration becomes comparable to other endoscopic methods (18). For this 85-year-old patient with significant comorbidities, the choice of Z-POEM was strategically justified. The minimally invasive nature of Z-POEM, combined with the enhanced safety profile from preserving the mucosa and the aim for a definitive, complete myotomy to prevent recurrence, represented the optimal balance of efficacy and safety for her specific clinical context (4, 13).

Although the mean hospital stay following Z-POEM is typically reported to be 3–5 days, with liquid diets often resuming within 24–48 h (4, 6, 7), our patient was discharged on postoperative day 12. Rather than reflecting a surgical complication, this prolonged hospitalization represents a deliberate, conservative, and individualized management strategy tailored to her high-risk geriatric profile. First, given the patient's advanced age (85 years), pre-existing malnutrition (as evidenced by a 5 kg weight loss and a hemoglobin level of 112.90 g/L), and cardiopulmonary comorbidities (emphysema and coronary sclerosis), standard rapid-recovery protocols were deemed inappropriately aggressive. We deliberately extended the nil per os (NPO) period to 72 h to guarantee secure mucosal healing. Furthermore, the extended administration of prophylactic antibiotics was specifically targeted to prevent aspiration pneumonia—a leading cause of mortality in frail elderly patients with dysphagia (21). Second, the 12-day hospitalization allowed for crucial comprehensive nutritional rehabilitation, cardiopulmonary monitoring, and a slow, supervised dietary step-up. Therefore, the length of stay in this context underscores an important clinical paradigm: in octogenarians, clinical success depends not only on technical execution but also on prioritizing conservative, patient-centered safety over the pursuit of the shortest possible hospitalization. Our case suggests that for patients aged 85 and older, the “clinical novelty” lies not in the surgical technique itself, but in the deliberate departure from standard fast-track protocols toward a “safety-first” individualized paradigm, which may be more appropriate for preventing postoperative pulmonary complications in the frailest populations.

This study has several limitations. Firstly, as a single-case report, its generalizability is limited, and larger cohort studies are needed to validate these findings. More importantly, a critical methodological limitation of this report is the complete absence of objective postoperative anatomical assessment. While follow-up endoscopy or barium esophagogram is the gold standard to confirm technical success, completeness of myotomy, and absence of recurrence, these could not be obtained because the 85-year-old patient and her family strongly refused further invasive or imaging tests. Consequently, our evaluation relies solely on clinical surrogate evidence. Nevertheless, in the context of high-risk geriatric care, the complete resolution of progressive dysphagia, the lack of complications, and a sustained 3.6 kg weight gain over one year provide highly valuable reference data, demonstrating that minimally invasive Z-POEM can achieve significant clinical and nutritional benefits even when anatomical confirmation is unfeasible.

## Data Availability

The original contributions presented in the study are included in the article/Supplementary Material, further inquiries can be directed to the corresponding author.
